# Image Encryption Using Quantum 3D Mobius Scrambling and 3D Hyper-Chaotic Henon Map

**DOI:** 10.3390/e25121629

**Published:** 2023-12-06

**Authors:** Ling Wang, Qiwen Ran, Junrong Ding

**Affiliations:** 1 School of Internet, Anhui University, Hefei 230039, China; 2 National Key Laboratory of Tunable Laser Technology, Harbin Institute of Technology, Harbin 150001, China

**Keywords:** quantum 3D Mobius scrambling, 3D hyper-chaotic Henon map, quantum circuits, quantum image encryption

## Abstract

In encryption technology, image scrambling is a common processing operation. This paper proposes a quantum version of the 3D Mobius scrambling transform based on the QRCI model, which changes not only the position of pixels but also the gray values. The corresponding quantum circuits are devised. Furthermore, an encryption scheme combining the quantum 3D Mobius transform with the 3D hyper-chaotic Henon map is suggested to protect the security of image information. To facilitate subsequent processing, the RGB color image is first represented with QRCI. Then, to achieve the pixel-level permutation effect, the quantum 3D Mobius transform is applied to scramble bit-planes and pixel positions. Ultimately, to increase the diffusion effect, the scrambled image is XORed with a key image created by the 3D hyper-chaotic Henon map to produce the encrypted image. Numerical simulations and result analyses indicate that our designed encryption scheme is secure and reliable. It offers better performance in the aspect of key space, histogram variance, and correlation coefficient than some of the latest algorithms.

## 1. Introduction

Based on the principles of quantum mechanics, quantum computing, which provides massive parallel computation [1], and unconditional security [2] for the data have been widely used in many information science fields. Quantum computing lays a solid foundation for the emergence and development of quantum information processing technology [3,4]. Since images are an important information transmission medium [5,6], how to process image information in quantum computers is a hotspot in research [7]. Quantum image processing has great significance to the security of images.

To store images by using qubits in quantum computers, researchers developed a great deal of representation models [8]. Qubit Lattice, as the first representation model, was put forward in 2003 [9]. Afterward, Real Ket, which allows using *n* qubits to represent an image of size $2^{n}\times 2^{n}$ was proposed [10]. In 2011, FRQI representation was designed [11], in which the image information was encoded as a superposition of quantum entangled states. Extending from FRQI, Zhang et al. presented NEQR representation [12]. It uses an entangled qubit sequence to encode grayscale information. After that, some other representation models were successively raised, including QUALPI [13], NAQSS [14], NCQI [15], GNEQR [16], FTQR [17], QIRHSI [18] and many more. Recently, Wang et al. devised the QRCI representation for storing RGB color images, which has a lower quantum cost [19].

Since quantum computing has enormous information-carrying capacity and strong computing power, many image encryption techniques making use of quantum computing have been suggested. Zhou et al. achieved an encryption algorithm with geometric transformations in 2013 [20]. In 2014, by means of restricted geometric and color transformations, Song et al. presented an encryption method for FRQI images [21]. Subsequently, the quantum realization circuit of the generalized Arnold transform was constructed and put to use in [22,23]. Gong et al. designed a novel encryption scheme by exploiting quantum XOR operations [24]. To solve the problem that some spatial domain transforms are periodic, an encryption algorithm by utilizing Fibonacci scrambling and geometric transform iteratively was presented [25]. In addition, Li et al. achieved an encryption method by exploiting phase-shift transform and Haar wavelet transform [26]. With the bit-level permutation operation, a cryptosystem for the NEQR model was proposed [27]. By utilizing image correlation decomposition, Zhang et al. proposed a quantum image encryption algorithm, which has a large key space [28]. Song et al. encrypted the QIRHSI image by using geometric transformation and intensity channel diffusion [29]. Lately, Liu et al. presented an independent bit-plane permutation, which was used to create a novel algorithm to encrypt quantum images [30]. Gao et al. developed an encryption technique based on quantum DNA coding and Hilbert scrambling operation [31].

Quantum image encryption using scrambling transforms is a widely used technique. Traditional algorithms like Arnold, Fibonacci, and Hilbert have been implemented using quantum circuits for image scrambling, as described in [32,33]. However, these algorithms have a limited range of scrambling and only disrupt the positions of pixels, leaving the distribution of pixel grayscales unchanged [34]. Therefore, it is crucial to explore newer and more efficient quantum scrambling algorithms that can be applied to quantum image encryption to enhance the security of cryptosystems. In this paper, a quantum image scrambling algorithm is designed based on the 3D Mobius transform to simultaneously change pixel locations and gray values, effectively eliminating correlation between adjacent pixels. To protect the security of quantum images, an encryption scheme is proposed that combines quantum 3D Mobius scrambling with a 3D hyper-chaotic Henon map. Firstly, the RGB color image is represented with QRCI. Then, to achieve the pixel-level permutation effect, the quantum 3D Mobius transform is applied to scramble bit-planes and pixel positions. Finally, to improve the security performance, quantum XOR operations controlled by the 3D hyper-chaotic Henon map are adopted to modify the color information of the scrambled image to obtain the ciphertext image. All of the quantum circuits utilized in the encryption procedure are developed. The main work of this study is summarized as below.

1. A quantum image-scrambling algorithm is created based on the 3D Mobius transform, which has a pixel-level scrambling effect and performs better than the quantum Arnold/Fibonacci transform.
2. A quantum image encryption scheme is suggested by combining quantum 3D Mobius scrambling with XOR diffusion. The quantum circuits for encryption operation are designed.
3. To obtain the desired encryption effect, the scrambling and diffusion operations are controlled by sequences generated by the 3D hyper-chaotic Henon map. The security of our encryption scheme is enhanced by the randomness and unpredictability of chaotic sequences.
4. Simulation results and comparative analysis demonstrate that our designed encryption scheme exhibits significant reliability and security.

The remainder of this paper is structured as follows. In Section 2, we briefly review the preliminary knowledge. Section 3 describes how to procure a quantum 3D Mobius scrambling algorithm in detail. The process of encryption and decryption is given in Section 4. Section 5 presents simulation results and comparative analysis. Finally, Section 6 concludes this paper.

## 2. Preliminaries

### 2.1. QRCI Image Representation Model

QRCI can represent a RGB color image of size $2^{n}\times 2^{n}$ by using only 2n+6 qubits [19], whose storage capacity is $2^{18}$ times higher than that of NCQI.

Supposing a $2^{n}\times 2^{n}$ RGB color image and each channel takes values within $\left[0,255\right]$, the corresponding QRCI is expressed as:

(1) $$\begin{array}{cc} |I\rangle & =\frac{1}{\sqrt{2^{2n+3}}}\sum_{L=0}^{2^{3}-1}\sum_{Y=0}^{2^{n}-1}\sum_{X=0}^{2^{n}-1}|C_{L}\left(Y,X\right)\rangle ⊗|LYX\rangle \\ & =\frac{1}{\sqrt{2^{2n+3}}}\sum_{L=0}^{2^{3}-1}\sum_{Y=0}^{2^{n}-1}\sum_{X=0}^{2^{n}-1}|R_{LYX}G_{LYX}B_{LYX}\rangle ⊗|LYX\rangle \end{array}$$

where $R_{LYX},G_{LYX},B_{LYX}\in \left\{0,1\right\}$ represent the values of three channels on the *L*-th bit-plane in position $\left(Y,X\right)$, respectively. $\left|L\rangle =\right|L_{2}L_{1}L_{0}\rangle$ denotes the bit-plane order and $\left|YX\rangle =\right|Y_{n-1}Y_{n-2}\ldots Y_{0}\rangle |X_{n-1}X_{n-2}\ldots X_{0}\rangle$ denotes the pixel position. The comparative analysis in reference [19] shows that QRCI requires fewer qubits compared with the other representation models.

### 2.2. Quantum Modules

Some basic quantum modules required in our algorithm are introduced in this subsection.

**Figure 1:**
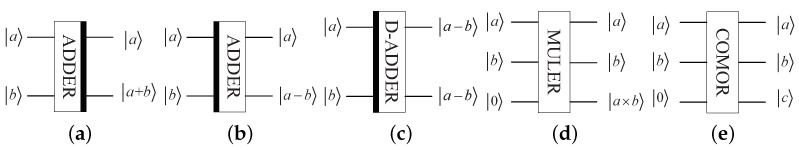
Basic quantum modules: (**a**) adder, (**b**) subtractor, (**c**) double-output adder, (**d**) multiplier, (**e**) comparator.

Quantum adder (ADDER) can calculate the sum of two numbers [35]. Its circuit is exhibited in Figure 1a.

If the black bar is adjusted to the left, Figure 1a will become a quantum subtractor. Figure 1b shows the corresponding circuit.

In addition, quantum double-output adder (D-ADDER) [36] and quantum multiplier (MULER) [37] are also exploited in this paper, whose circuits are displayed in Figure 1c and Figure 1d, respectively.

Quantum comparator (COMOR) is implemented to show the comparison result of two numbers, i.e., c=0, when b≥a; otherwise, c=1 [38]. The corresponding circuit is shown in Figure 1e.

### 2.3. 3D Hyper-Chaotic Henon Map

**Figure 2:**
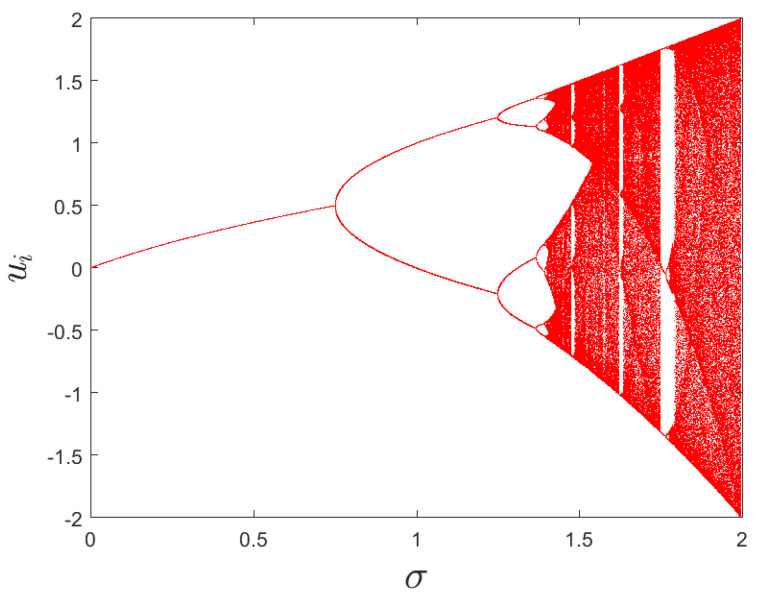
The bifurcation diagram of *u*-sequence.

**Figure 3:**
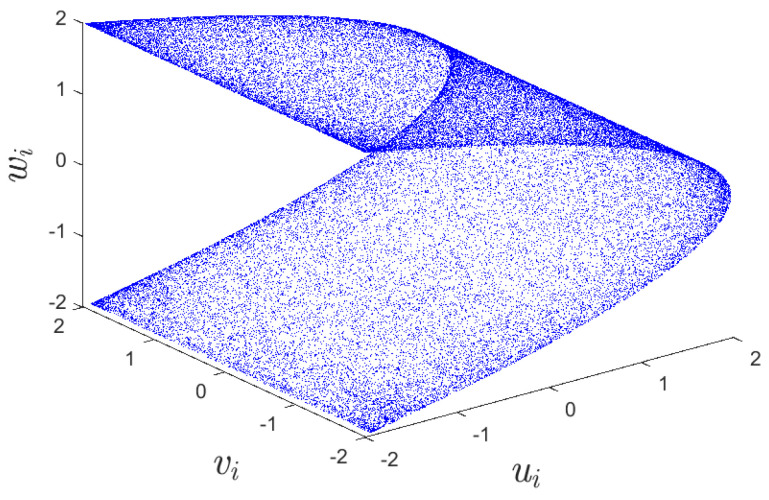
The phase diagram of 3D hyper-chaotic Henon map.

The mathematical expression of 3D hyper-chaotic Henon map is:

(2) $$\left\{\begin{array}{c} u_{i+1}=\sigma -v_{i}^{2}-\rho w_{i}\\ v_{i+1}=u_{i}\\ w_{i+1}=v_{i} \end{array}\right.$$

where *u*, *v* and *w* denote three variables, while σ and ρ are two control parameters. When σ and ρ are 1.99, 0.001, respectively, map (2) exhibits a hyper-chaotic behavior [39]. The initial values are set to $u_{0}=0$, $v_{0}=0$, and $w_{0}=0.1$. Figure 2 and Figure 3 display the bifurcation diagram and phase diagram of the 3D hyper-chaotic Henon map, respectively.

As the hyper-chaotic maps have more complicated dynamic behaviors than 1D chaotic maps, this paper adopts map (2) to create the sequences controlling the encryption operations. The randomness and unpredictability of chaotic sequences will improve the encryption effect.

## 3. Three-Dimensional (3D) Mobius Quantum Image-Scrambling Algorithm

### 3.1. Two-Dimensional (2D) Mobius Transform

The Mobius band is a topological transform in continuous space; that is, the rectangular band is folded reversely and then the vertices are overlapped in pairs. To apply this topological transform to image scrambling, the image can be regarded as a rectangular Mobius band consisting of discrete dot arrays. Suppose the matrix of an image with size M×N is represented as $A={\left[a_{ij}\right]}_{M\times N}$. When the image is folded reversely along the horizontal direction, the *i*-th row and the $\left(M+1-i\right)$-th row $\left(i=1,2,\cdots ,M\right)$ correspond to each other. Likewise, when the image is folded reversely along the vertical direction, the *j*-th column and the $\left(N+1-j\right)$-th column $\left(j=1,2,\cdots ,N\right)$ correspond to each other. Pairwise corresponding rows or columns form a circle. By performing shift operation on it, the image-scrambling model based on the Mobius band can be deduced, where the count of the cycle shift operation can be regarded as the image-scrambling parameters.

Assuming there are two sequences $\left\{r\left(1\right),r\left(2\right),\ldots ,r\left(M\right)\right\}$, $\left\{c\left(1\right),c\left(2\right),\ldots ,c\left(N\right)\right\}$, for ∀i,j, they satisfy $r\left(i\right)=r\left(M-i+1\right)\in \left\{1,2,\ldots ,N\right\}$, $c\left(j\right)=c\left(N-j+1\right)\in \left\{1,2,\ldots ,M\right\}$; then, the discrete expression of spatial domain image scrambling based on the 2D Mobius transform is as shown below.

For the horizontal direction folding, $S_{x}\left(i,j\right)$ is as:

(3) $$S_{x}\left(i,j\right)=\left\{\begin{array}{c} \left(M-i+1,N-r\left(i\right)+j\right),\;1\leq j\leq r\left(i\right)\\ \left(i,j-r\left(i\right)\right),\;r\left(i\right)<j\leq N \end{array}\right.$$

where $r\left(i\right)$ is the shift count of the *i*-th row.

For the vertical direction folding, $S_{y}\left(i,j\right)$ is as:

(4) $$S_{y}\left(i,j\right)=\left\{\begin{array}{c} \left(M-c\left(j\right)+i,N-j+1\right),\;1\leq i\leq c\left(j\right)\\ \left(i-c\left(j\right),j\right),\;c\left(j\right)<i\leq M \end{array}\right.$$

where $c\left(j\right)$ is the shift count of the *j*-th column.

The scrambled image $S\left(A\right)$ can be obatined after the original image *A* is successively transformed once by (3) and (4). In order to obtain a better scrambling effect, the scrambling operation can be performed many times.

The inverse 2D Mobius transform can be realized by executing the following equations:

(5) $$S_{y}^{-1}\left(i,j\right)=\left\{\begin{array}{c} \left(i+c\left(j\right)-M,N-j+1\right),\;M-c\left(j\right)<i\leq M\\ \left(i+c\left(j\right),j\right),\;1\leq i\leq M-c\left(j\right) \end{array}\right.$$

(6) $$S_{x}^{-1}\left(i,j\right)=\left\{\begin{array}{c} \left(M-i+1,j+r\left(i\right)-N\right),\;N-r\left(i\right)<j\leq N\\ \left(i,j+r\left(i\right)\right),\;1\leq j\leq N-r\left(i\right) \end{array}\right.$$

### 3.2. Three-Dimensional (3D) Mobius Scrambling Algorithm

Two-dimensional (2D) Mobius transform can be expanded to the 3D Mobius transform. Suppose there is a three-dimensional cube $A={\left[a_{ijl}\right]}_{W\times H\times L}$. Three matrices ${\left[r_{1}\left(j,l\right)\right]}_{H\times L}$, ${\left[r_{2}\left(i,l\right)\right]}_{W\times L}$ and ${\left[r_{3}\left(i,j\right)\right]}_{W\times H}$ are used to control the shift counts, and for ∀i,j,l, they satisfy $r_{1}\left(j,l\right)=r_{1}\left(H-j+1,L-l+1\right)\in \left\{1,2,\cdots ,W\right\}$, $r_{2}\left(i,l\right)=r_{2}\left(W-i+1,L-l+1\right)\in \left\{1,2,\cdots ,H\right\}$, $r_{3}\left(i,j\right)=r_{3}\left(W-i+1,H-j+1\right)\in \left\{1,2,\cdots ,L\right\}$. The corresponding three scrambling operations based on the 2D Mobius transform are as follows.

For the *x*-axis direction folding, the expression of discrete transform $S_{x}\left(i,j,l\right)$ is as follows:

(7) $$S_{x}\left(i,j,l\right)=\left\{\begin{array}{c} \left(W-r_{1}\left(j,l\right)+i,H-j+1,L-l+1\right),\;1\leq i\leq r_{1}\left(j,l\right)\\ \left(i-r_{1}\left(j,l\right),j,l\right),\;r_{1}\left(j,l\right)<i\leq W \end{array}\right.$$

where $r_{1}\left(j,l\right)$ is the shift count.

For the *y*-axis direction folding, the expression of discrete transform $S_{y}\left(i,j,l\right)$ is as follows:

(8) $$S_{y}\left(i,j,l\right)=\left\{\begin{array}{c} \left(W-i+1,H-r_{2}\left(i,l\right)+j,L-l+1\right),\;1\leq j\leq r_{2}\left(i,l\right)\\ \left(i,j-r_{2}\left(i,l\right),l\right),\;r_{2}\left(i,l\right)<j\leq H \end{array}\right.$$

where $r_{2}\left(i,l\right)$ is the shift count.

Likewise, for the *z*-axis direction folding, the expression of discrete transform $S_{z}\left(i,j,l\right)$ is as follows:

(9) $$S_{z}\left(i,j,l\right)=\left\{\begin{array}{c} \left(W-i+1,H-j+1,L-r_{3}\left(i,j\right)+l\right),\;1\leq l\leq r_{3}\left(i,j\right)\\ \left(i,j,l-r_{3}\left(i,j\right)\right),\;r_{3}\left(i,j\right)<l\leq L \end{array}\right.$$

where $r_{3}\left(i,j\right)$ is the shift count.

Calculating Equations (7)–(9) in order; then, the result of 3D Mobius scrambling can be obtained. The scrambling algorithm can be performed as many times as needed.

The inverse 3D Mobius transform can be realized by executing the following equations in order:

(10) $$S_{z}^{-1}\left(i,j,l\right)=\left\{\begin{array}{c} \left(W-i+1,H-j+1,l+r_{3}\left(i,j\right)-L\right),\;L-r_{3}\left(i,j\right)<l\leq L\\ \left(i,j,l+r_{3}\left(i,j\right)\right),\;1\leq l\leq L-r_{3}\left(i,j\right) \end{array}\right.$$

(11) $$S_{y}^{-1}\left(i,j,l\right)=\left\{\begin{array}{c} \left(W-i+1,j+r_{2}\left(i,l\right)-H,L-l+1\right),\;H-r_{2}\left(i,l\right)<j\leq H\\ \left(i,j+r_{2}\left(i,l\right),l\right),\;1\leq j\leq H-r_{2}\left(i,l\right) \end{array}\right.$$

(12) $$S_{x}^{-1}\left(i,j,l\right)=\left\{\begin{array}{c} \left(i+r_{1}\left(j,l\right)-W,H-j+1,L-l+1\right),\;W-r_{1}\left(j,l\right)<i\leq W\\ \left(i+r_{1}\left(j,l\right),j,l\right),\;1\leq i\leq W-r_{1}\left(j,l\right) \end{array}\right.$$

### 3.3. The Quantum Circuit of 3D Mobius Scrambling

In this paper, the QRCI model is adopted, which can be viewed as a 3D image model, where the three dimensions are the *X*-axis, *Y*-axis, and *L*-axis, respectively. Therefore, we consider scrambling a QRCI image shown in Equation (1) by using the 3D Mobius transform. It should be noted that $X,Y\in \left[0,2^{n}-1\right]$ and $L\in \left[0,2^{3}-1\right]$. Assuming $S=S_{L}S_{Y}S_{X}$, quantum 3D Mobius scrambling transform $M_{b}$ can be constructed as follows:

(13) $$M_{b}=I^{⊗3}⊗S=I^{⊗3}⊗\left(S_{L}S_{Y}S_{X}\right)$$

By performing $M_{b}$ on the QRCI image |I〉, the scrambled image $|I_{M_{b}}\rangle$ can be obtained:

(14) $$\begin{array}{cc} |I_{M_{b}}\rangle & =M_{b}|I\rangle \\ & =\left(I^{⊗3}⊗S\right)\frac{1}{\sqrt{2^{2n+3}}}\sum_{L=0}^{2^{3}-1}\sum_{Y=0}^{2^{n}-1}\sum_{X=0}^{2^{n}-1}|C_{L}\left(Y,X\right)\rangle ⊗|LYX\rangle \\ & =\frac{1}{\sqrt{2^{2n+3}}}\sum_{L=0}^{2^{3}-1}\sum_{Y=0}^{2^{n}-1}\sum_{X=0}^{2^{n}-1}|C_{L}\left(Y,X\right)\rangle ⊗S_{L}S_{Y}S_{X}\left(|LYX\rangle \right)\\ & =\frac{1}{\sqrt{2^{2n+3}}}\sum_{L=0}^{2^{3}-1}\sum_{Y=0}^{2^{n}-1}\sum_{X=0}^{2^{n}-1}|C_{L}\left(Y,X\right)\rangle ⊗|L^{*}Y^{*}X^{*}\rangle \end{array}$$

where $S_{X}$, $S_{Y}$ and $S_{L}$ perform the following functions:

(15) $$\begin{array}{cc} & |L^{\prime }Y^{\prime }X^{\prime }\rangle =S_{X}|LYX\rangle \\ = & \left\{\begin{array}{c} |2^{3}-1-L\rangle |2^{n}-1-Y\rangle |2^{n}-U\left(L,Y\right)+X\rangle ,\;0\leq X<U\left(L,Y\right)\\ \left|L\rangle |Y\rangle \right|X-U\left(L,Y\right)\rangle ,\;U\left(L,Y\right)\leq X\leq 2^{n}-1 \end{array}\right. \end{array}$$

(16) $$\begin{array}{cc} & |L^{\prime }Y^{\prime }X^{\prime }\rangle =S_{Y}|LYX\rangle \\ = & \left\{\begin{array}{c} |2^{3}-1-L\rangle |2^{n}-V\left(L,X\right)+Y\rangle |2^{n}-1-X\rangle ,\;0\leq Y<V\left(L,X\right)\\ \left|L\rangle \right|Y-V\left(L,X\right)\rangle |X\rangle ,\;V\left(L,X\right)\leq Y\leq 2^{n}-1 \end{array}\right. \end{array}$$

(17) $$\begin{array}{cc} & |L^{\prime }Y^{\prime }X^{\prime }\rangle =S_{L}|LYX\rangle \\ = & \left\{\begin{array}{c} |2^{3}-W\left(Y,X\right)+L\rangle |2^{n}-1-Y\rangle |2^{n}-1-X\rangle ,\;0\leq L<W\left(Y,X\right)\\ |L-W\left(Y,X\right)\rangle |Y\rangle |X\rangle ,\;W\left(Y,X\right)\leq L\leq 2^{3}-1 \end{array}\right. \end{array}$$

where $U\left(L,Y\right)$, $V\left(L,X\right)$ and $W\left(Y,X\right)$ denote the shift counts of folding along the *X*-axis, *Y*-axis and *L*-axis, respectively. For ∀X,Y,L, they satisfy $U\left(L,Y\right)=U\left(2^{3}-1-L,2^{n}-1-Y\right)\in \left\{0,1,\ldots ,2^{n}-1\right\}$, $V\left(L,X\right)=V\left(2^{3}-1-L,2^{n}-1-X\right)\in \left\{0,1,\ldots ,2^{n}-1\right\}$, $W\left(Y,X\right)=W\left(2^{n}-1-Y,2^{n}-1-X\right)\in \left\{0,1,\ldots ,2^{3}-1\right\}$.

According to Equation (13), the quantum 3D Mobius scrambling algorithm can be implemented by three sub-operations, i.e., $S_{X}$, $S_{Y}$ and $S_{L}$. For the sub-operation $S_{X}$, it can be implemented as follows. First, the subfunction, when $0\leq X<U\left(L,Y\right)$, is implemented:

(18) $$P|LYX\rangle =|2^{3}-1-L\rangle |2^{n}-1-Y\rangle |2^{n}-U\left(L,Y\right)+X\rangle$$

After that, the result obtained from Equation (18) is transformed by the following formula:

(19) $$Q|LYX\rangle =|2^{3}-1-L\rangle |2^{n}-1-Y\rangle |X-2^{n}\rangle$$

As a result, the composite of *P* and *Q* is equivalent to the subfunction when $U\left(L,Y\right)\leq X\leq 2^{n}-1$:

(20) $$QP|LYX\rangle =\left|L\rangle |Y\rangle \right|X-U\left(L,Y\right)\rangle$$

**Figure 4:**
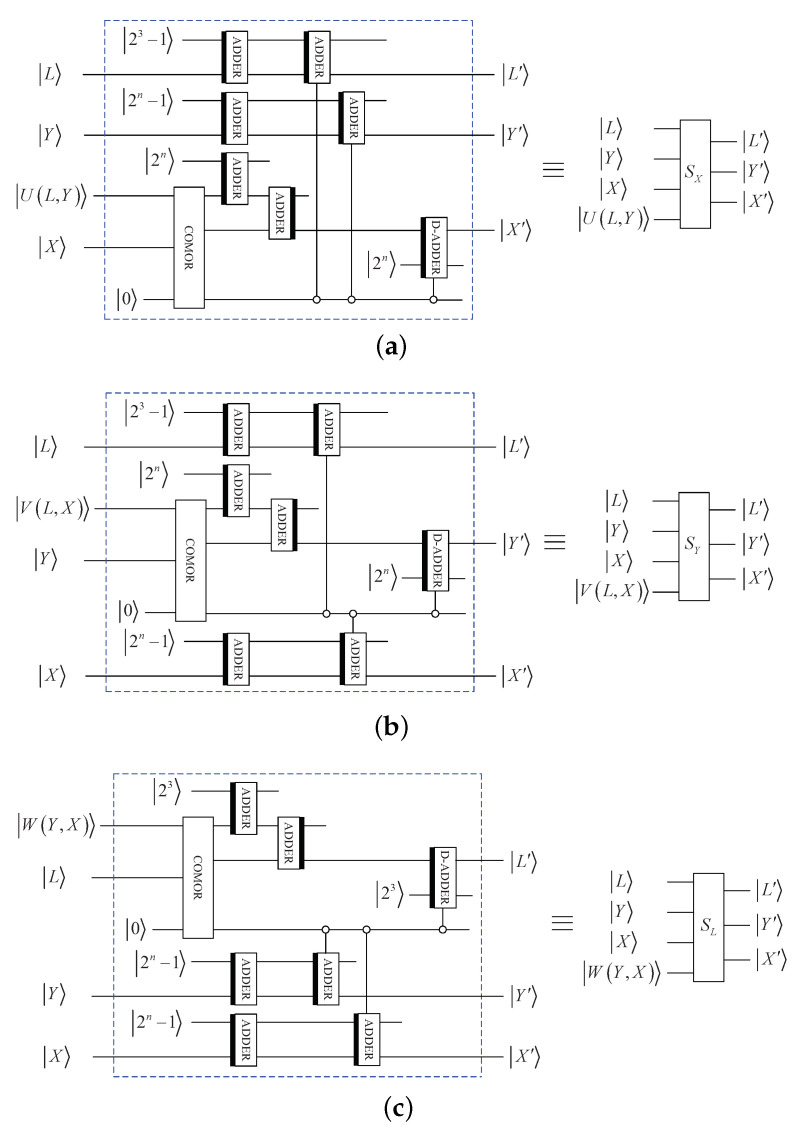
Elementary quantum circuits: (**a**) $S_{X}$. (**b**) $S_{Y}$. (**c**) $S_{L}$.

By using some basic quantum modules, Figure 4a shows the circuit of $S_{X}$. Similarly, Figure 4b,c shows the circuits of $S_{Y}$ and $S_{L}$, respectively.

**Figure 5:**
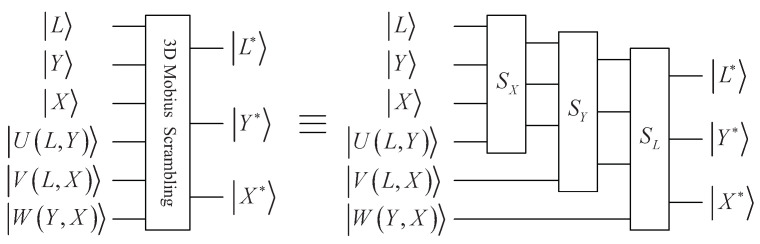
Quantum circuit for 3D Mobius scrambling transform.

Above all, the whole quantum circuit of 3D Mobius scrambling is shown in Figure 5.

Inverse 3D Mobius scrambling is needed to recover the original image. Supposing $S^{-1}=S_{X}^{-1}S_{Y}^{-1}S_{L}^{-1}$, quantum inverse 3D Mobius scrambling transform $M_{b}^{-1}$ can be constructed as:

(21) $$M_{b}^{-1}=I^{⊗3}⊗S^{-1}=I^{⊗3}⊗\left(S_{X}^{-1}S_{Y}^{-1}S_{L}^{-1}\right)$$

Through applying the transform $M_{b}^{-1}$ to $|I_{M_{b}}\rangle$, the original QRCI image can be gained:

(22) $$\begin{array}{cc} |I\rangle & =M_{b}^{-1}|I_{M_{b}}\rangle \\ & =\left(I^{⊗3}⊗S^{-1}\right)\frac{1}{\sqrt{2^{2n+3}}}\sum_{L=0}^{2^{3}-1}\sum_{Y=0}^{2^{n}-1}\sum_{X=0}^{2^{n}-1}|C_{L}\left(Y,X\right)\rangle ⊗|L^{*}Y^{*}X^{*}\rangle \\ & =\frac{1}{\sqrt{2^{2n+3}}}\sum_{L=0}^{2^{3}-1}\sum_{Y=0}^{2^{n}-1}\sum_{X=0}^{2^{n}-1}|C_{L}\left(Y,X\right)\rangle ⊗S_{X}^{-1}S_{Y}^{-1}S_{L}^{-1}\left(|L^{*}Y^{*}X^{*}\rangle \right)\\ & =\frac{1}{\sqrt{2^{2n+3}}}\sum_{L=0}^{2^{3}-1}\sum_{Y=0}^{2^{n}-1}\sum_{X=0}^{2^{n}-1}|C_{L}\left(Y,X\right)\rangle ⊗|LYX\rangle \end{array}$$

where $S_{L}^{-1}$, $S_{Y}^{-1}$ and $S_{X}^{-1}$ perform the following functions:

(23) $$\begin{array}{cc} & |L^{\prime }Y^{\prime }X^{\prime }\rangle =S_{L}^{-1}|LYX\rangle \\ = & \left\{\begin{array}{c} |L+W\left(Y,X\right)-2^{3}\rangle |2^{n}-1-Y\rangle |2^{n}-1-X\rangle ,\;2^{3}-W\left(Y,X\right)\leq L\leq 2^{3}-1\\ |L+W\left(Y,X\right)\rangle |Y\rangle |X\rangle ,\;0\leq L<2^{3}-W\left(Y,X\right) \end{array}\right. \end{array}$$

(24) $$\begin{array}{cc} & |L^{\prime }Y^{\prime }X^{\prime }\rangle =S_{Y}^{-1}|LYX\rangle \\ = & \left\{\begin{array}{c} |2^{3}-1-L\rangle |Y+V\left(L,X\right)-2^{n}\rangle |2^{n}-1-X\rangle ,\;2^{n}-V\left(L,X\right)\leq Y\leq 2^{n}-1\\ \left|L\rangle \right|Y+V\left(L,X\right)\rangle |X\rangle ,\;0\leq Y<2^{n}-V\left(L,X\right) \end{array}\right. \end{array}$$

(25) $$\begin{array}{cc} & |L^{\prime }Y^{\prime }X^{\prime }\rangle =S_{X}^{-1}|LYX\rangle \\ = & \left\{\begin{array}{c} |2^{3}-1-L\rangle |2^{n}-1-Y\rangle |X+U\left(L,Y\right)-2^{n}\rangle ,\;2^{n}-U\left(L,Y\right)\leq X\leq 2^{n}-1\\ \left|L\rangle |Y\rangle \right|X+U\left(L,Y\right)\rangle ,\;0\leq X<2^{n}-U\left(L,Y\right) \end{array}\right. \end{array}$$

For the sub-operation $S_{L}^{-1}$, it can be implemented in two steps. The first step is to implement the subfunction when $0\leq L<2^{3}-W\left(Y,X\right)$:

(26) $$P^{\prime }\left|LYX\rangle =\right|L+W\left(Y,X\right)\rangle |Y\rangle |X\rangle$$

The second step is to modify the produced result by utilizing the following formula:

(27) $$Q^{\prime }\left|LYX\rangle =\right|L-2^{3}\rangle |2^{n}-1-Y\rangle |2^{n}-1-X\rangle$$

Therefore, when $2^{3}-W\left(Y,X\right)\leq L\leq 2^{3}-1$, the corresponding subfunction is equivalent to the composite of $P^{\prime }$ and $Q^{\prime }$ as below:

(28) $$Q^{\prime }P^{\prime }\left|LYX\rangle =\right|L+W\left(Y,X\right)-2^{3}\rangle |2^{n}-1-Y\rangle |2^{n}-1-X\rangle$$

**Figure 6:**
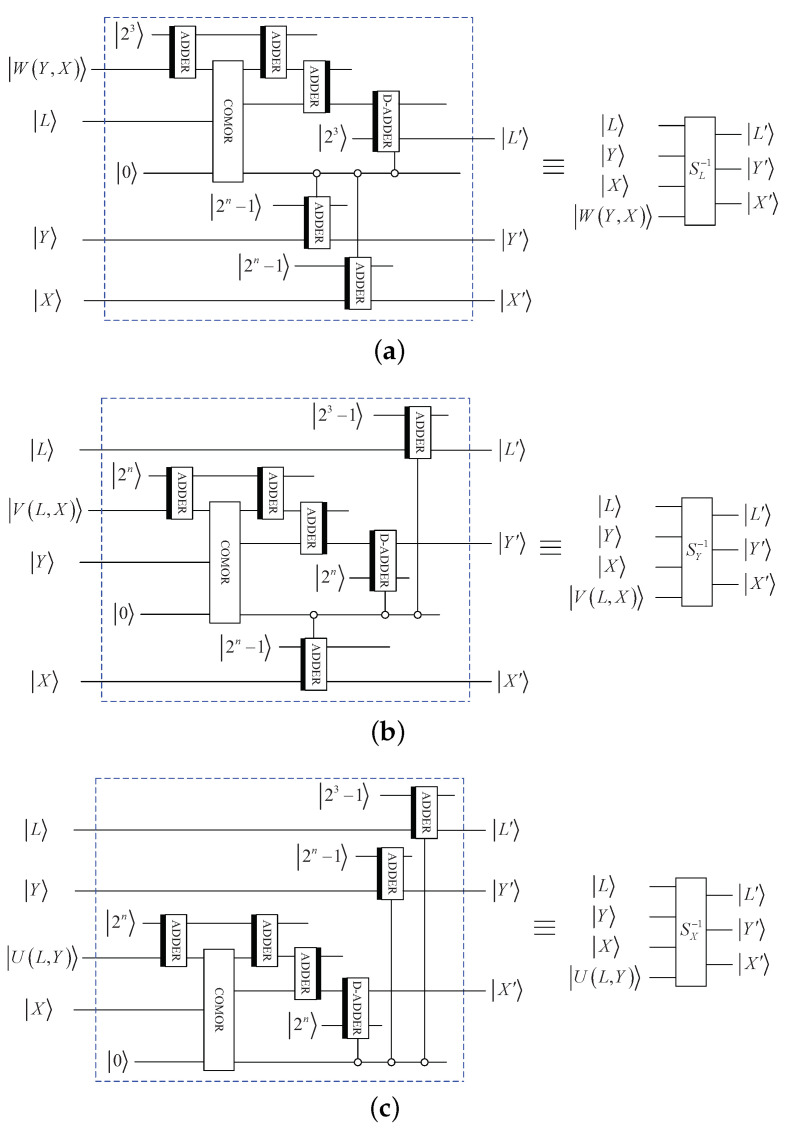
Elementary quantum circuits: (**a**) $S_{L}^{-1}$. (**b**) $S_{Y}^{-1}$. (**c**) $S_{X}^{-1}$.

The quantum circuit of sub-operation $S_{L}^{-1}$ is depicted in Figure 6a. In the same way, the quantum circuits of $S_{Y}^{-1}$ and $S_{X}^{-1}$ are depicted in Figure 6b,c.

**Figure 7:**
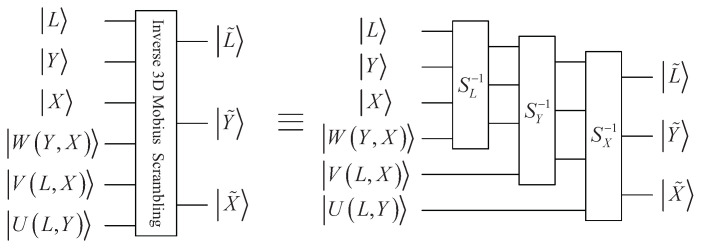
Quantum circuit for inverse 3D Mobius transform.

The whole circuit for quantum inverse 3D Mobius scrambling transform is shown in Figure 7.

### 3.4. Scrambling Result and Anti-Attack Ability Analysis

**Figure 8:**
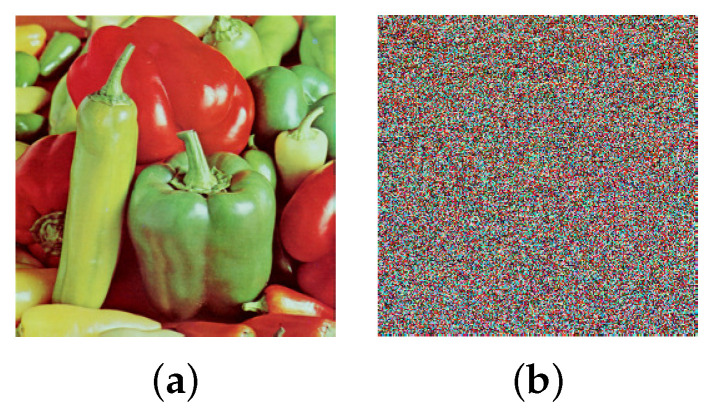
Scrambling results: (**a**) Peppers, (**b**) scrambled Peppers.

To verify the scrambling result of the 3D Mobius transform, a 256×256 color image of peppers shown in Figure 8a is used for testing. Three matrices that control the shift counts are randomly selected. The scrambled image after applying the 3D Mobius transform one time is shown in Figure 8b. It demonstrates that the 3D Mobius scrambling transform is effective and can visually hide the information about the image.

**Figure 9:**
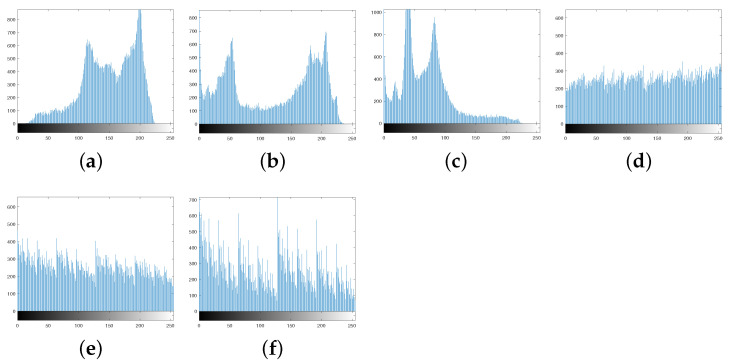
Histograms: (**a**) R channel of Peppers. (**b**) G channel of Peppers. (**c**) B channel of Peppers. (**d**) R channel of scrambled Peppers. (**e**) G channel of scrambled Peppers. (**f**) B channel of scrambled Peppers.

**Figure 10:**
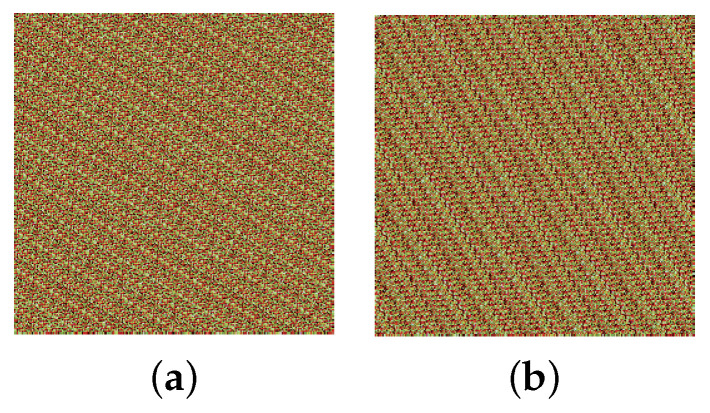
Scrambling results of Arnold/Fibonacci transform: (**a**) Arnold. (**b**) Fibonacci.

**Figure 11:**
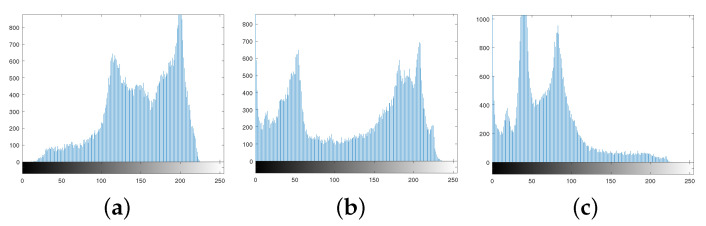
Histograms of using Arnold/Fibonacci transform: (**a**) R channel. (**b**) G channel. (**c**) B channel.

The histogram distributions are displayed in Figure 9. Figure 10a,b show the scrambled images after 100 iterations of the Arnold and Fibonacci transforms, respectively. The corresponding RGB histograms are displayed in Figure 11. The 3D Mobius scrambling transform differs from the Arnold/Fibonacci transform in that it changes both the position of pixels and the distribution of gray values in the histogram. This is because it simultaneously scrambles bit-planes and pixel positions. As a result, the 3D Mobius scrambling transform alters the statistical properties of the original image to a significant extent.

To further estimate the scrambling performance of the 3D Mobius transform, the correlation of adjacent pixels is considered. The formula for calculating the coefficient used to evaluate this correlation is:

(29) $$CC=\frac{\sum_{i=1}^{H}\left(x_{i}-\bar{x}\right)\left(y_{i}-\bar{y}\right)}{\sqrt{\sum_{i=1}^{H}{\left(x_{i}-\bar{x}\right)}^{2}\sum_{i=1}^{H}{\left(y_{i}-\bar{y}\right)}^{2}}}$$

where $x_{i}$ and $y_{i}$ denote two neighboring pixel values. $\bar{x}=\frac{1}{H}\sum_{i=1}^{H}x_{i}$ and $\bar{y}=\frac{1}{H}\sum_{i=1}^{H}y_{i}$ are the expectation values. The CC values of Peppers in horizontal, vertical, and diagonal directions are listed in Table 1. It is shown that the correlation of adjacent pixels has been significantly reduced.

Hou et al. proposed a quantum image-scrambling algorithm based on a discrete Baker map, which could be implemented by swapping qubits [34]. The CC values of scrambled Peppers derived from the Baker map are compiled in Table 2. The results show that the correlation between adjacent pixels decreases with the increase in scrambling times. After 16 times of scrambling, the adjacent pixels are almost no longer correlated. It can be seen from Table 1 and Table 2 that the 3D Mobius scrambling could weaken the correlation better since it has more scrambling parameters.

If the scrambling algorithm is open, assume that the attackers do not know the key matrices and make a brute-force attack. Since $U\left(L,Y\right)\in \left[0,2^{n}-1\right]$, $V\left(L,X\right)\in \left[0,2^{n}-1\right]$, $W\left(Y,X\right)\in \left[0,2^{3}-1\right]$, in the 3D Mobius transform, for each row transform in the X-axis direction, the probability of cracking success is $\frac{1}{2^{n}}$. Therefore, for the entire X-axis direction; the probability of cracking success is ${2^{n}}^{-2^{n}\times 2^{3}}$. The coupling of the *X*-axis, *Y*-axis, and *Z*-axis makes it even more difficult to crack, the probability of cracking success is ${2^{n}}^{-2^{n}\times 2^{3}}{2^{n}}^{-2^{n}\times 2^{3}}{2^{3}}^{-2^{n}\times 2^{n}}$. Thus, the 3D Mobius transform has a relatively good resistance to brute-force attacks.

Consequently, the quantum 3D Mobius scrambling is employed as an encryption means in the encryption scheme to be raised below.

## 4. Encryption and Decryption Scheme

### 4.1. Encryption Scheme

**Figure 12:**
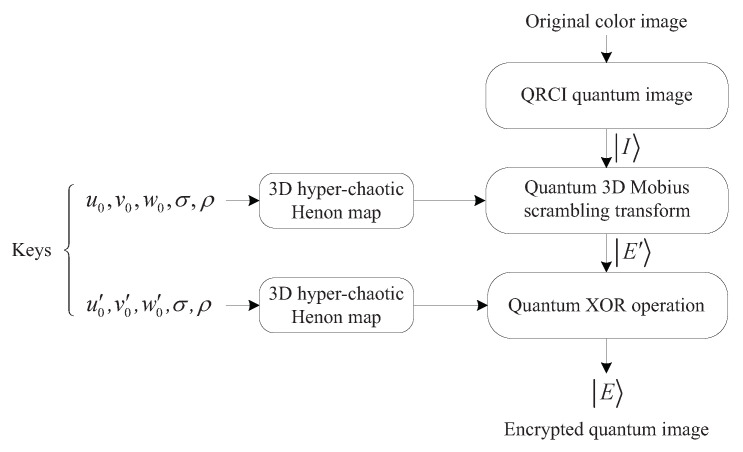
The encryption procedure.

Combining 3D Mobius scrambling with the 3D hyper-chaotic Henon map, our proposed quantum image encryption scheme includes five steps. Figure 12 exhibits the encryption procedure.

Input: A classical RGB color image with size $2^{n}\times 2^{n}$.

Keys: Two sets of initial values, $u_{0},v_{0},w_{0}$, $u_{0}^{\prime },v_{0}^{\prime },w_{0}^{\prime }$ and two control parameters σ, ρ.

Output: The final ciphertext image |E〉.

**Step 1.** The original RGB color image is represented with QRCI as follows:

(30) $$|I\rangle =\frac{1}{\sqrt{2^{2n+3}}}\sum_{L=0}^{2^{3}-1}\sum_{Y=0}^{2^{n}-1}\sum_{X=0}^{2^{n}-1}|R_{LYX}G_{LYX}B_{LYX}\rangle ⊗|LYX\rangle$$

**Step 2.** This step generates three matrices that control the quantum 3D Mobius scrambling transform.

**Figure 13:**
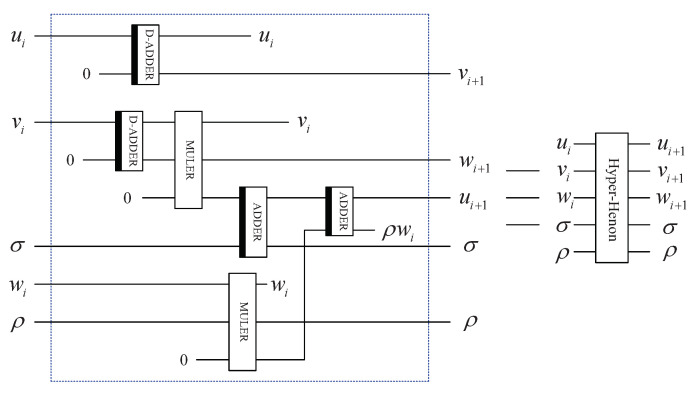
Quantum circuit for implementing one iteration.

According to Equation (2), six computation modules are required for each iteration of the 3D hyper-chaotic Henon map, and the circuit is presented in Figure 13.

**Figure 14:**
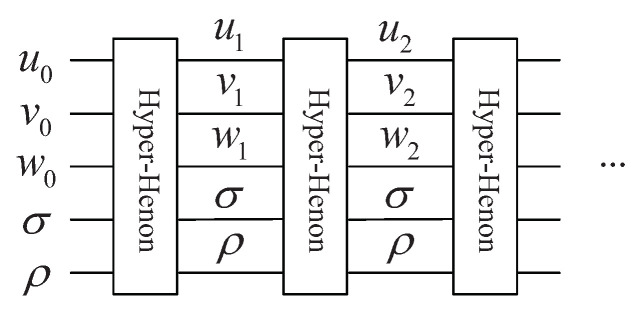
The complete quantum circuit for 3D hyper-chaotic Henon map.

By utilizing the complete circuit shown in Figure 14, Equation (2) is iterated $\frac{2^{n}\times 2^{n}}{2}+k$ times with the first set of initial values $u_{0},v_{0}$, and $w_{0}$, where *k* is a positive integer. In general, $k=2\times 10^{4}$. The sequences are taken from the k+1 iteration, since the randomness of the beginning part may not be good enough. The constructed sequences $\left\{u_{k+1},u_{k+2},\cdots ,u_{k+2^{2n-1}}\right\}$, $\left\{v_{k+1},v_{k+2},\cdots ,v_{k+2^{2n-1}}\right\}$ and $\left\{w_{k+1},w_{k+2},\cdots ,w_{k+2^{2n-1}}\right\}$ cannot be used directly. They are transformed into integer sequences as shown below:

(31) $$\left\{\begin{array}{c} U_{i}=floor\left(u_{i+k+1}\times 10^{14}\right)\;\mathrm{mod}\;2^{n}\\ V_{i}=floor\left(v_{i+k+1}\times 10^{14}\right)\;\mathrm{mod}\;2^{n}\\ W_{j}=floor\left(w_{j+k+1}\times 10^{14}\right)\;\mathrm{mod}\;2^{3} \end{array}\right.$$

where $i=0,1,\cdots ,\frac{2^{3}\times 2^{n}}{2}-1$, $j=0,1,\cdots ,\frac{2^{n}\times 2^{n}}{2}-1$.

Then, the three integer sequences are extended to ${\left[U\left(L,Y\right)\right]}_{2^{3}\times 2^{n}}$, ${\left[V\left(L,X\right)\right]}_{2^{3}\times 2^{n}}$, ${\left[W\left(Y,X\right)\right]}_{2^{n}\times 2^{n}}$, and for ∀X,Y,L, they satisfy $U\left(L,Y\right)=U\left(2^{3}-1-L,2^{n}-1-Y\right)\in \left\{0,1,\cdots ,2^{n}-1\right\}$, $V\left(L,X\right)=V\left(2^{3}-1-L,2^{n}-1-X\right)\in \left\{0,1,\cdots ,2^{n}-1\right\}$, $W\left(Y,X\right)=W\left(2^{n}-1-Y,2^{n}-1-X\right)\in \left\{0,1,\cdots ,2^{3}-1\right\}$. The obtained integer matrices will be used to control the shift counts.

**Step 3.** By using the obtained three matrices and the quantum 3D Mobius scrambling transform $M_{b}$ designed in Section 3.3, the scrambled image $|E^{\prime }\rangle$ will be produced after carrying out the 3D Mobius transform on original image |I〉:

(32) $$\begin{array}{cc} |E^{\prime }\rangle & =M_{b}|I\rangle \\ & =\left(I^{⊗3}⊗S\right)\frac{1}{\sqrt{2^{2n+3}}}\sum_{L=0}^{2^{3}-1}\sum_{Y=0}^{2^{n}-1}\sum_{X=0}^{2^{n}-1}|R_{LYX}G_{LYX}B_{LYX}\rangle ⊗|LYX\rangle \\ & =\frac{1}{\sqrt{2^{2n+3}}}\sum_{L=0}^{2^{3}-1}\sum_{Y=0}^{2^{n}-1}\sum_{X=0}^{2^{n}-1}|R_{LYX}G_{LYX}B_{LYX}\rangle ⊗S\left(|LYX\rangle \right)\\ & =\frac{1}{\sqrt{2^{2n+3}}}\sum_{L=0}^{2^{3}-1}\sum_{Y=0}^{2^{n}-1}\sum_{X=0}^{2^{n}-1}|R_{LYX}G_{LYX}B_{LYX}\rangle ⊗|L^{*}Y^{*}X^{*}\rangle \end{array}$$

where $|L^{*}\rangle$, $|Y^{*}\rangle$, and $|X^{*}\rangle$ can be obtained by calculating Equations (15)–(17) in order.

Different from some classical two-dimensional scrambling transforms, for instance, Arnold/Fibonacci transform [32], 3D Mobius scrambling transform not only disrupts the pixel positions but also disrupts the order of bit-planes. Hence, after the image is transformed, the distribution of its pixel values will be changed, which can improve the security of the cryptosystem. Moreover, the randomness and unpredictability of the parameters in three matrices will make it much harder to decipher the 3D Mobius transform.

**Step 4.** In this step, we exploit the 3D hyper-chaotic Henon map to construct sequences controlling quantum XOR operations. Similar to step 2, the map shown in Equation (2) is iterated $2^{n}\times 2^{n}+k$ times with the second set of initial values $u_{0}^{\prime },v_{0}^{\prime }$, and $w_{0}^{\prime }$. Three chaotic sequences $\left\{u_{k+1}^{\prime },u_{k+2}^{\prime },\cdots ,u_{k+2^{2n}}^{\prime }\right\}$, $\left\{v_{k+1}^{\prime },v_{k+2}^{\prime },\cdots ,v_{k+2^{2n}}^{\prime }\right\}$ and $\left\{w_{k+1}^{\prime },w_{k+2}^{\prime },\cdots ,w_{k+2^{2n}}^{\prime }\right\}$ can be generated. Then, they are calculated as shown below:

(33) $$\left\{\begin{array}{c} U_{i}^{\prime }=floor\left(u_{i+k+1}^{\prime }\times 10^{14}\right)\;\mathrm{mod}\;2^{8}\\ V_{i}^{\prime }=floor\left(v_{i+k+1}^{\prime }\times 10^{14}\right)\;\mathrm{mod}\;2^{8}\\ W_{i}^{\prime }=floor\left(w_{i+k+1}^{\prime }\times 10^{14}\right)\;\mathrm{mod}\;2^{8} \end{array}\right.$$

where $i=0,1,\cdots ,2^{2n}-1$, $U_{i}^{\prime },andV_{i}^{\prime },W_{i}^{\prime }\in \left\{0,1,\cdots ,255\right\}$.

After that, $\left\{U_{i}^{\prime }\right\}$, $\left\{V_{i}^{\prime }\right\}$, $\left\{W_{i}^{\prime }\right\}$ are stored as an RGB color key image |K〉. The QRCI representation of |K〉 is as follows:

(34) $$|K\rangle =\frac{1}{\sqrt{2^{2n+3}}}\sum_{L=0}^{2^{3}-1}\sum_{Y=0}^{2^{n}-1}\sum_{X=0}^{2^{n}-1}|U_{LYX}^{\prime }V_{LYX}^{\prime }W_{LYX}^{\prime }\rangle ⊗|LYX\rangle$$

**Step 5.** To further prevent the extraction of original data by unauthorized persons, quantum XOR operations are implemented between the scrambled image $|E^{\prime }\rangle$ and the key image |K〉 pixel by pixel, and the final encrypted image |E〉 will be obtained.

**Figure 15:**
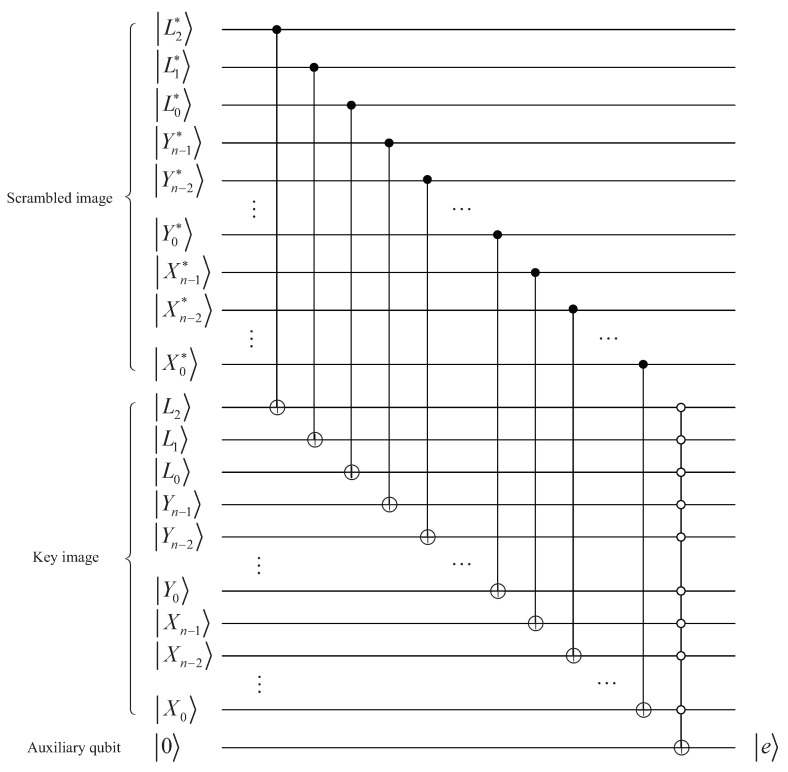
Quantum circuit for synchronizing positions and bit-planes.

Of course, in order to implement the XOR operation, it should be noted that the positions and bit-planes of $|E^{\prime }\rangle$ must be equal to that of |K〉. The quantum circuit to synchronize the positions and bit-planes is given in Figure 15, and they are equal when output |e〉=|1〉.

**Figure 16:**
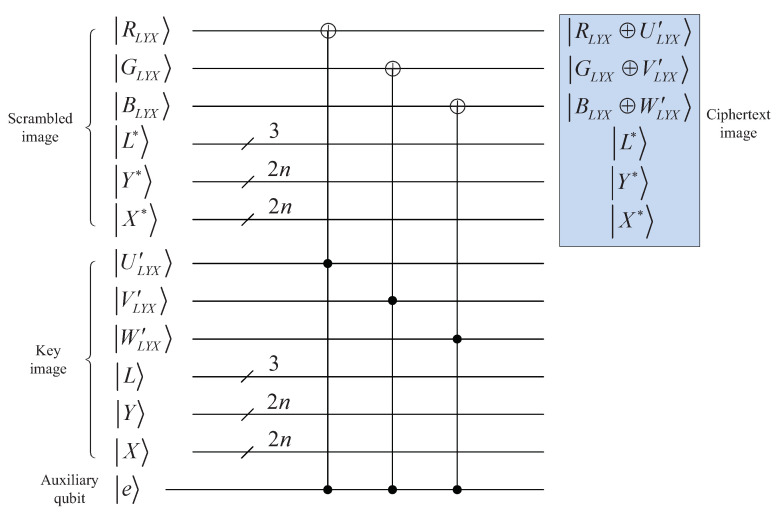
Quantum circuit to implement XOR operation.

The circuit implementing quantum XOR operations is given in Figure 16, in which the output is the final ciphertext image |E〉.

### 4.2. Decryption Scheme

The specific decryption steps are described as below.

**Step 1.** Taking advantage of correct keys $u_{0}^{\prime },v_{0}^{\prime },andw_{0}^{\prime }$, three hyper-chaotic integer sequences $\left\{U_{0}^{\prime },U_{1}^{\prime },\cdots ,U_{2^{2n}-1}^{\prime }\right\}$, $\left\{V_{0}^{\prime },V_{1}^{\prime },\cdots ,V_{2^{2n}-1}^{\prime }\right\}$ and $\left\{W_{0}^{\prime },W_{1}^{\prime },\cdots ,W_{2^{2n}-1}^{\prime }\right\}$ could be obtained with Step 4 in the encryption process. They are stored in a key image |K〉 based on QRCI representation.

**Step 2.** The received ciphertext image |E〉 is XORed with |K〉 to obtain the scrambled image $|E^{\prime }\rangle$.

**Step 3.** According to Step 2 in the encryption process, three hyper-chaotic integer matrices ${\left[U\left(L,Y\right)\right]}_{2^{3}\times 2^{n}}$, ${\left[V\left(L,X\right)\right]}_{2^{3}\times 2^{n}}$ and ${\left[W\left(Y,X\right)\right]}_{2^{n}\times 2^{n}}$ which control the shift counts can be constructed.

**Step 4.** Using the produced three control matrices, the decrypted quantum image represented with the QRCI model can be obtained by executing the inverse quantum 3D Mobius scrambling transform on $|E^{\prime }\rangle$.

**Step 5.** The classical image is recovered with quantum measurements. Since QRCI representation stores an image as the superposition of qubit basic states, the classical image can be accurately recovered. In reference [40], the measurement of recovering classical information from the QRCI quantum state was given exhaustively. No detailed explanation of it will be given in this article.

## 5. Numerical Simulation and Comparative Analysis

The simulations are conducted on a classical computer due to the lack of quantum devices. The selected test images are four 256×256 RGB color images, i.e., Lena, Baboon, Splash, and House. According to Equation (2), the map will exhibit a hyper-chaotic behavior when σ and ρ are 1.99 and 0.001, respectively. The initial values are set as $u_{0}=1,v_{0}=0.1,w_{0}=0$ and $u_{0}^{\prime }=1,v_{0}^{\prime }=1,w_{0}^{\prime }=1$.

### 5.1. Visual Effects

**Figure 17:**
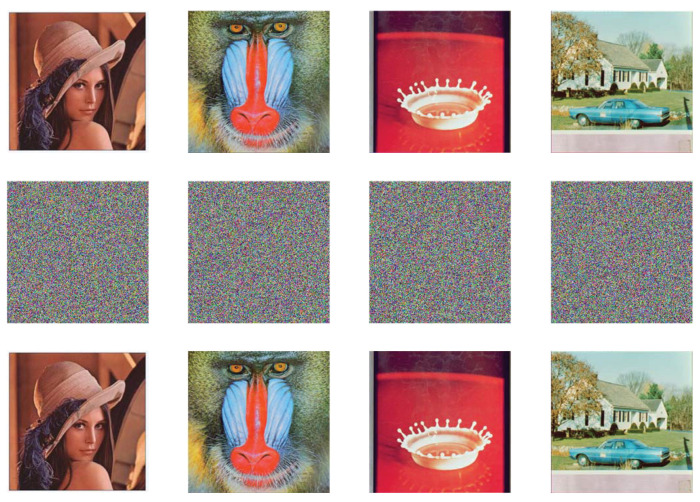
Simulation results.

Figure 17 shows the simulation results of encryption and decryption. Therein, the four subgraphs in the first row are original images, while the middle row and the last row are encrypted images and decrypted images with correct keys, respectively. Obviously, no meaningful information can be identified from the encrypted images. This verifies that our encryption technique can provide visual protection for the original image.

### 5.2. Histogram Analysis

**Figure 18:**
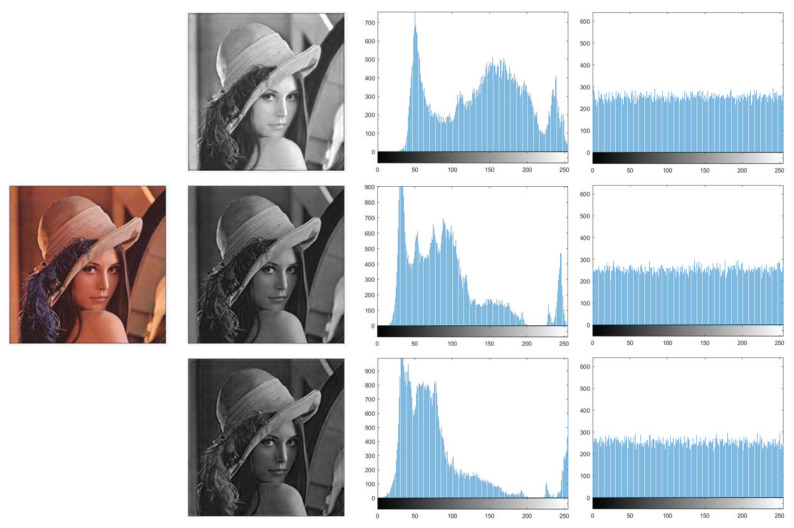
Histograms of Lena.

**Figure 19:**
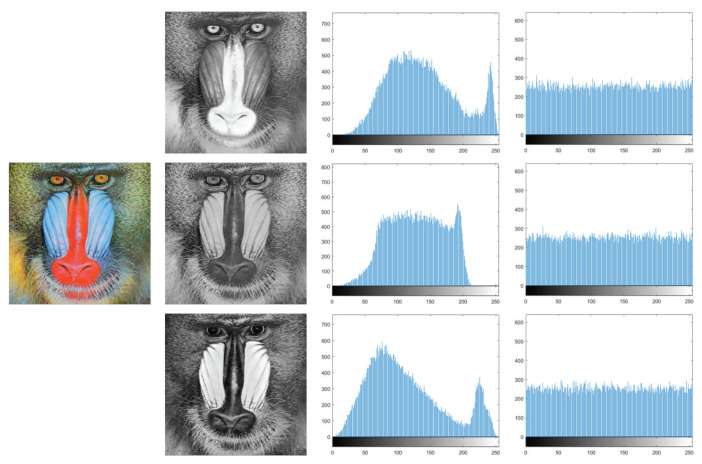
Histograms of Baboon.

The information characteristics of an image can be intuitively reflected by histograms. For a satisfactory ciphertext image, its pixel values must be uniformly distributed [41]. In our proposed encryption scheme, the combination of scrambling and diffusion operations enables a uniform distribution of pixel values from 0 to 255. Lena and Baboon are chosen as test images, and the histogram distributions of RGB three channels are illustrated in Figure 18 and Figure 19, respectively. It manifests that the original image has an uneven histogram distribution, while the histogram of the encrypted image obtained by our scheme becomes uniform.

Therefore, the attacker could not obtain the original image by analyzing the histogram distribution.

The histogram variance [30] which can quantitatively measure the degree of change in pixel distribution is defined as follows:

(35) $$HV=\frac{\sum_{i=0}^{255}\sum_{j=0}^{255}\frac{1}{2}{\left(\gamma_{i}-\gamma_{j}\right)}^{2}}{256^{2}}$$

where $\gamma_{i}$ and $\gamma_{j}$ are the number of pixels with grayscale values of *i* and *j*, respectively. Table 3 lists the computed variance values. Compared with the original images, the HV values of encrypted images are much lower, which is observed in Table 3. Hence, our image encryption method is resistant to statistical attacks based on histogram variance analysis.

### 5.3. Encryption Quality Analysis

(1) Uniform histogram deviation

Uniform histogram deviation (UHD) is commonly used to estimate image cryptosystem encryption quality [42]. UHD is calculated as follows:

(36) $$UHD=\frac{\sum_{\delta =0}^{255}\left|O-O_{\delta }\right|}{M\times N}$$

where M×N is the size of the image. The histogram of the ciphertext image under index δ is represented by $O_{\delta }$ and *O* is a uniform histogram. A smaller UHD value indicates a higher encryption quality. The UHD values of the ciphertext images are compiled in Table 4. Apparently, the UHD values of four encrypted images are all close to 0. Therefore, our proposed scheme exhibits acceptable encryption quality.

(2) Irregular deviation

Irregular deviation (ID) is also frequently introduced to evaluate the quality of encrypted images [42].

(37) $$ID=\sum_{\delta =0}^{255}\left|R_{\delta }-\bar{R}\right|$$

where $R_{\delta }$ refers to the absolute histogram difference between the initial image and its encrypted version, $\bar{R}=1/256\sum_{\delta =0}^{255}R_{\delta }$. The larger the ID value, the higher the quality of the encrypted image. The ID values in Table 4 are all sufficiently large, indicating that our scheme can produce high-quality encrypted images.

(3) Maximum deviation

The maximum deviation (MD) [42] is adopted for describing the extreme error between the original image and its ciphertext. MD can be expressed as shown below:

(38) $$MD=max\left|P\left(i,j\right)-C\left(i,j\right)\right|$$

where $P\left(i,j\right)$ and $C\left(i,j\right)$ denote the pixel values in the original image and its corresponding encrypted one, respectively. As the MD value increases, the encryption quality also improves. From the results in Table 4, it can be seen that the MD values are very large. Therefore, the encryption quality of our scheme is acceptable.

### 5.4. Correlation Analysis

**Figure 20:**
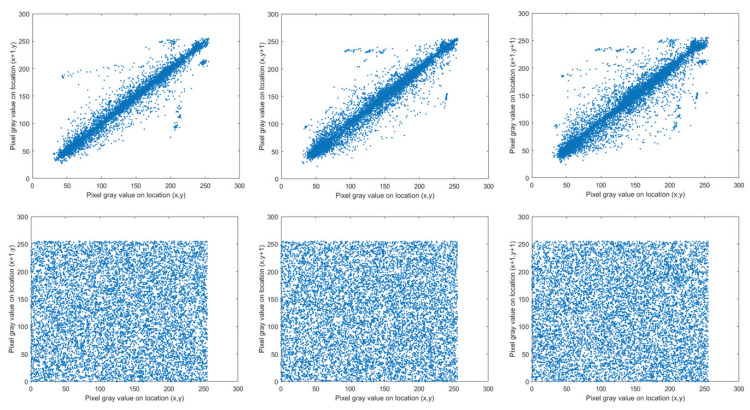
The correlation distributions.

A good encryption technique should break the correlation between neighboring pixels [43]. In the suggested encryption scheme, the 3D Mobius scrambling transform possesses the capacity to weaken the correlation coefficient among adjacent pixels. The R channel of Lena is selected for testing, and 10,000 pairs of neighboring pixels are randomly chosen in all three directions. Figure 20 represents the correlation distribution results. Therein, the three subgraphs in the first row show the correlations of Lena horizontally, vertically, and diagonally, respectively, and those of the encrypted Lena are shown in the three subgraphs in the second row. The ciphertext image has a uniform pixel distribution, which demonstrates that the proposed encryption technique considerably decreases the correlation.

The data in Table 5 reflect the specific CC values. The CC values of encrypted images are minimized to be close to 0, meaning attackers cannot crack our algorithm relying on correlation analysis.

### 5.5. Information Entropy

A crucial metric for assessing the randomness of ciphertext images is information entropy. Mathematically, it is calculated as shown below:

(39) $$IE=-\sum_{i=0}^{255}P\left(i\right)\log_{2}P\left(i\right)$$

where $P\left(i\right)$ denotes the appearance frequency of gray value *i*. In general, the perfect IE value is 8. The utilization of 3D Mobius scrambling and XOR coding controlled by chaotic sequences can significantly improve the randomness of encrypted images. The precise IE values of original and encrypted images are provided in Table 6. From the results, the IE values of encrypted images are all approximate to 8. Therefore, our encryption method can effectively resist entropy attacks.

### 5.6. Spectrum Analysis

**Figure 21:**
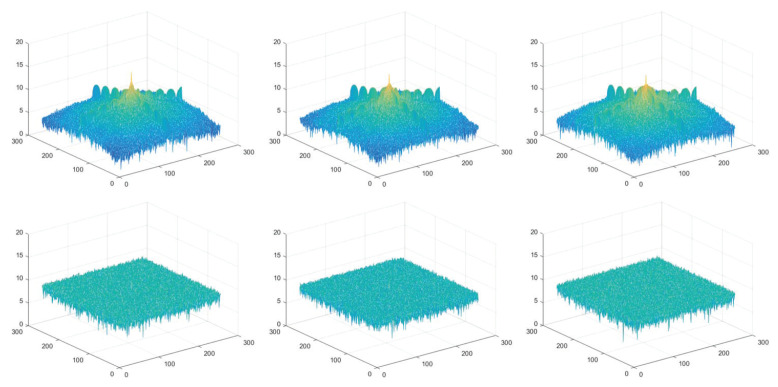
The spectrum distributions.

The Fourier spectrums of Lena are visualized in Figure 21, in which the three subgraphs in the first row show the spectral distributions of RGB channels of the original Lena, and those of encrypted Lena are displayed in the three subgraphs in the second row. It is evident that the spectrums of ciphertext images have a uniform distribution. In the meantime, the spectrum of each channel is similar to each other. Thus, our proposed encryption technique can stand up to spectrum attacks.

### 5.7. Key Sensitivity and Key Space

**Figure 22:**
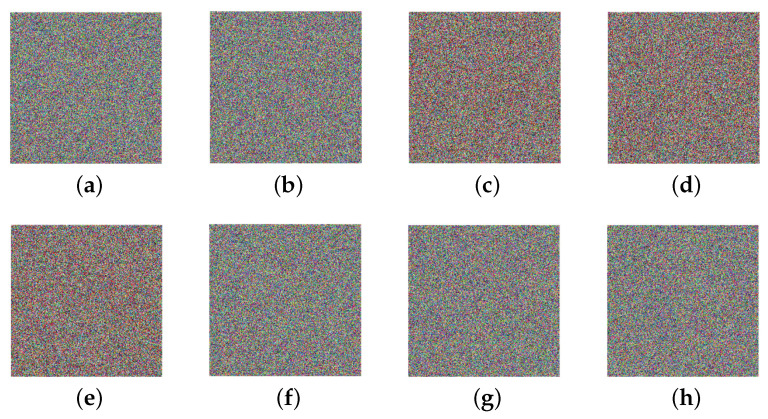
Decrypted images with incorrect keys: (**a**) $\sigma +10^{-15}$. (**b**) $\rho +10^{-18}$. (**c**) $u_{0}+10^{-15}$. (**d**) $v_{0}+10^{-15}$. (**e**) $w_{0}+10^{-12}$. (**f**) $u_{0}^{\prime }+10^{-15}$. (**g**) $v_{0}^{\prime }+10^{-15}$. (**h**) $w_{0}^{\prime }+10^{-13}$.

In this study, all of the scrambling and diffusion operations are controlled by sequences generated by the 3D hyper-chaotic Henon map. Two control parameters and two sets of initial values for this map, i.e., σ, ρ, $u_{0},v_{0},w_{0}$, and $u_{0}^{\prime },v_{0}^{\prime },w_{0}^{\prime }$ are taken as encryption keys. Since the chaotic systems are particularly sensitive to the initial values, the presented cryptosystem achieves a strong key sensitivity. The Lena image is opted for testing the sensitivity of respective keys. Figure 22 gives the results decrypted by using keys with a subtle change.

Visibly, noise-like images would be obtained with incorrect keys. Therefore, the decrypted image is available only if all the keys are correct, which indicates this scheme is sensitive to the keys.

In accordance with the sensitivity experiments described above, the total key space is up to $10^{118}$, which is about equal to $2^{392}$. Therefore, the brute-force attack does not work on deciphering our encryption scheme.

### 5.8. Noise Attack and Cutting Attack

**Figure 23:**
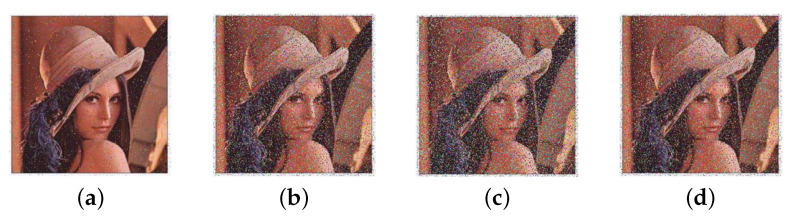
Decrypted images with various noise intensities: (**a**) 0.25. (**b**) 0.5. (**c**) 0.75. (**d**) 1.

Suppose a ciphertext image is contaminated with noise:

(40) $$E^{\prime }=E+sG$$

where *E* and $E^{\prime }$ represent ciphertext images before and after contamination, respectively. *G* is Gaussian noise with zero mean and standard deviation, and *s* is the noise strength. The ciphertext image of Lena is used for testing, Figure 23 depicts the corresponding decrypted results when *s* takes the value of 0.25, 0.5, 0.75, and 1. The results indicate that our encryption algorithm is somewhat robust to noise attacks.

**Figure 24:**
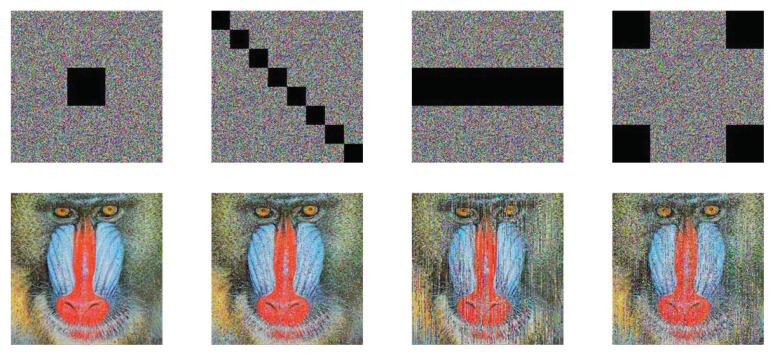
The cutting attack results.

To test the performance of our scheme to combat cutting attacks, regions of different sizes are cut from the encrypted Baboon. Figure 24 presents the corresponding decrypted versions. As can be seen, although some details are broken, the primary information is still available. The simulation results allow us to make clear that our proposed algorithm could resist cutting attacks to some degree.

### 5.9. Computational Complexity

The proposed quantum image encryption scheme’s computational complexity is primarily related to the quantum 3D Mobius scrambling transform and quantum XOR operation. The quantum 3D Mobius scrambling transform is made up of basic quantum modules, including ADDER, D-ADDER, MULER, and COMOR. The complexity of each module can be reduced to O(n), as stated by [36,38]. Therefore, the computational complexity of the quantum 3D Mobius scrambling transform is $O\left(n\right)$. In Figure 15 and Figure 16, there are $\left(2n+3\right)$ CNOT gates, one $\left(2n+3\right)$-CNOT gate and three Toffoli gates. Since one *t*-CNOT gate can be decomposed into $2\left(t-1\right)$ Toffoli gates and one CNOT gate, one Toffoli gate can be realized by six CNOT gates [44]. Thus, the quantum XOR operation involves $\left(26n+46\right)$ CNOT gates. Consequently, the whole computational complexity of our proposed encryption scheme is $O\left(n\right)$. While in the similar classical image encryption algorithm, all operations are performed on each pixel, so that the complexity is up to $O\left(2^{2n}\right)$. It is clear that the proposed quantum image encryption scheme has lower computational complexity than its classical counterparts.

### 5.10. Performance Comparison

This subsection compares our encryption scheme with some latest quantum image encryption algorithms [29,40,45,46]. The comparison contents include the key space, histogram variance, correlation coefficient, and information entropy. Table 7, Table 8, Table 9 and Table 10 summarize the comparison results.

In Table 7, the key space of our scheme is much larger than those of [29,40,45,46] and greatly exceeds the minimal limit of $2^{100}$. Thus, our proposed scheme has a significant advantage. It is hard for attackers to decipher the encrypted images by brute-force attack unless they have entirely correct keys. As can be observed from Table 8, the histogram variance values of the encrypted images generated with our proposed scheme are smaller than those in [29,40,45], which illustrates the superior performance of our image encryption scheme. Table 9 presents the comparison concerning the correlation coefficient. Obviously, the correlation coefficients of the encrypted images yielded by our proposed scheme reach 0 more tightly than those in [29,46], and they are close to the values in [40,45], which implies that our encryption operations greatly weaken the correlation among adjacent pixels. From Table 10, the information entropies with our encryption scheme are near to the values in [45,46], while they are smaller than the values in [29,40]. Nonetheless, the information entropy values obtained using our scheme are already very approximate to the ideal value of 8 bits. Hence, the proposed image encryption scheme can effectively thwart the information entropy attack. Overall, the above performance comparisons confirm the effectiveness and merits of the proposed quantum image encryption scheme.

## 6. Conclusions

In this paper, a quantum image-scrambling algorithm based on the 3D Mobius transform is investigated and its quantum realization circuit is developed, which changes not only the position of pixels but also the gray values. After that, by combining 3D Mobius scrambling with a 3D hyper-chaotic Henon map, an encryption scheme for a QRCI quantum image is proposed. In the permutation stage, the 3D Mobius transform is adopted to scramble bit-planes and pixel positions. In the diffusion stage, the 3D hyper-chaotic Henon map is employed to further improve the scheme’s performance, and the generated hyper-chaotic sequences are utilized to control quantum XOR operations. The 3D Mobius transform has more parameters such as the shift counts for folding along different axes than the Arnold/Fibonacci transform. The introduction of a 3D hyper-chaotic Henon map improves our encryption scheme greatly in terms of randomness and unpredictability. This ensures our scheme has good security. Additionally, the entire image encryption process could be implemented in quantum computers, thereby breaking the limitations of classical computers. Simulation results and comparative analysis show the validity and reliability of our proposed encryption scheme. In the future, we hope to define a scrambling operation with better results than the 3D Mobius transform and apply it to quantum image encryption.

## Figures and Tables

**Table 1:** Adjacent pixel correlation coefficient.

Peppers	Original			Scrambled		
	**Horizontal**	**Vertical**	**Diagonal**	**Horizontal**	**Vertical**	**Diagonal**
R	0.9704	0.9646	0.9400	0.0114	0.0103	0.0119
G	0.9740	0.9698	0.9470	−0.0091	0.0062	−0.0077
B	0.9645	0.9534	0.9261	0.0046	0.0065	0.0068

**Table 2:** Adjacent pixel correlation coefficient of scrambled Peppers in [34].

Scrambled Peppers	Horizontal	Vertical	Diagonal
1 time	0.9704	0.8369	0.7654
3 times	0.6275	0.2013	0.0708
16 times	0.0724	0.0724	0.0176
96 times	0.0285	0.0170	0.0129

**Table 3:** Results of HV values.

Image	Original			Encrypted		
	**R**	**G**	**B**	**R**	**G**	**B**
Lena	$2.6138\times 10^{4}$	$6.4845\times 10^{4}$	$9.1647\times 10^{4}$	238.2891	254.7422	261.3984
Baboon	$2.8890\times 10^{4}$	$4.2994\times 10^{4}$	$2.6968\times 10^{4}$	278.2109	241.6875	265.4297
Splash	$1.5125\times 10^{5}$	$1.7515\times 10^{5}$	$3.9784\times 10^{5}$	240.6875	266.0469	255.7109
House	$4.8896\times 10^{4}$	$8.2361\times 10^{4}$	$6.2631\times 10^{4}$	236.0391	228.6563	279.2969

**Table 4:** Results of UHD, ID, and MD values.

Encrypted Image		R	G	B
Lena	UHD	0.0508	0.0391	0.0859
	ID	19843	22798	32022
	MD	253	249	254
Baboon	UHD	0.0156	0.0547	0.0469
	ID	17562	11876	18396
	MD	250	233	249
Splash	UHD	0.0391	0.0547	0.0352
	ID	35399	40164	66062
	MD	240	255	240
House	UHD	0.0820	0.0742	0.0313
	ID	25663	27041	32412
	MD	233	243	246

**Table 5:** Results of CC values.

Image	Horizontal		Vertical		Diagonal	
	**Original**	**Encrypted**	**Original**	**Encrypted**	**Original**	**Encrypted**
Lena (R)	0.9718	0.0043	0.9668	−0.0026	0.9343	0.0017
Lena (G)	0.9644	0.0041	0.9534	0.0055	0.9139	0.0041
Lena (B)	0.9538	−0.0018	0.9490	−0.0025	0.9113	−0.0043
Baboon (R)	0.9270	−0.0031	0.9462	−0.0038	0.9117	0.0011
Baboon (G)	0.8450	−0.0036	0.8689	−0.0011	0.7952	0.0023
Baboon (B)	0.9113	0.0029	0.9207	0.0012	0.8709	0.0068
Splash (R)	0.9971	−0.0021	0.9861	0.0019	0.9857	−0.0051
Splash (G)	0.9805	0.0049	0.9690	0.0041	0.9516	0.0052
Splash (B)	0.9719	−0.0056	0.9675	−0.0036	0.9485	0.0012
House (R)	0.9354	0.0034	0.9369	−0.0040	0.8811	0.0033
House (G)	0.9300	0.0013	0.9164	−0.0019	0.8575	0.0018
House (B)	0.9586	0.0050	0.9608	0.0049	0.9138	0.0052

**Table 6:** Results of IE values.

Image	Original			Encrypted		
	**R**	**G**	**B**	**R**	**G**	**B**
Lena	7.6353	7.2778	7.0656	7.9974	7.9972	7.9971
Baboon	7.6058	7.3581	7.6665	7.9970	7.9973	7.9971
Splash	6.9417	6.9045	6.0601	7.9974	7.9971	7.9972
House	7.4025	7.2317	7.4280	7.9974	7.9975	7.9969

**Table 7:** Key space comparison.

Algorithm	Ours	Ref. [29]	Ref. [40]	Ref. [45]	Ref. [46]
Key space	$10^{118}\approx 2^{392}$	$10^{83}$	$2^{177}$	$10^{112}$	$10^{60}$

**Table 8:** Histogram variance comparison.

Image	Red	Green	Blue
Baboon (512×512)	$4.8324\times 10^{5}$	$7.0917\times 10^{5}$	$4.5314\times 10^{5}$
Enc-Baboon	931.3	907.2	1175.6
Ref. [29]	1333.1		
Ref. [45]	1130.8		
Splash (512×512)	$2.4061\times 10^{6}$	$2.7794\times 10^{6}$	$6.3912\times 10^{6}$
Enc-Splash	1038.7	1048.6	1049.4
Ref. [29]	1164.3		
Peppers (512×512)	$9.1085\times 10^{5}$	$7.6877\times 10^{5}$	$1.6001\times 10^{6}$
Enc-Peppers	1142.2	1031.8	950.3
Ref. [45]	4155.3		
Lena (256×256)	$2.6138\times 10^{4}$	$6.4845\times 10^{4}$	$9.1647\times 10^{4}$
Enc-Lena	238.3	254.7	261.4
Ref. [40]	242.8	262.1	284.9
Ref. [45]	273.3		

**Table 9:** Correlation coefficient comparison.

Image	Horizontal	Vertical	Diagonal
Enc-Peppers (R)	0.0044	−0.0033	0.0026
Enc-Peppers (G)	0.0021	−0.0002	0.0036
Enc-Peppers (B)	0.0048	0.0022	−0.0019
Ref. [29]	−0.0067	−0.0038	0.0063
Ref. [46]	−0.0036	−0.0539	0.0455
Enc-Lena (R)	0.0043	−0.0026	0.0017
Enc-Lena (G)	0.0041	0.0055	0.0041
Enc-Lena (B)	−0.0018	−0.0025	−0.0043
Ref. [40] (R)	0.0029	−0.0033	0.0019
Ref. [40] (G)	−0.0025	−0.0059	0.0013
Ref. [40] (B)	−0.0063	0.0046	−0.0036
Ref. [45] (R)	−0.0006	−0.0049	0.0070
Ref. [45] (G)	0.0025	−0.0051	0.0020
Ref. [45] (B)	0.0046	0.0019	0.0047

**Table 10:** Information entropy comparison.

Image	Red	Green	Blue
Enc-Baboon	7.9970	7.9973	7.9971
Ref. [29]	7.9991		
Ref. [40]	7.9993	7.9993	7.9993
Ref. [45]	7.9972	7.9969	7.9971
Enc-Peppers	7.9972	7.9972	7.9968
Ref. [45]	7.9970	7.9965	7.9962
Ref. [46]	7.9973		

## Data Availability

The data are contained within the article.
